# Annotations

**Published:** 1905-12-23

**Authors:** 


### ANNOTATIONS.

### Parliament and the Profession.

This Journal, of course, has nothing to do with politics as such. But at a General Election it becomes important that any influence we possess should be cast in favour of the fullest possible representation of the profession, by the best men, in Parliament. There can be no doubt that more medical practitioners are needed in the House of Commons, which requires in these days the presence amongst its members of a goodly number of scientists and doctors who are also men of affairs. Amongst the candidates for London Constituencies is a medical graduate who has given remarkable proof of his ability in business and has shown by years of splendid service, that in affairs he has few equals. Sir William Collins has been a member of the L.C.C. for some fourteen years and was its Chairman in 1897-98, when he made his mark as an able administrator and tactician. He was appointed the first Chairman of the London Education Committee last year, a position of supreme importance and difficulty, the duties of which he has discharged with great ability and success. He has been one of the most active members of the Senate of the University of London, is a member of the Executive and of the Council of King Edward's Hospital Fund, and also one of the Honorary Secretaries of the League of Mercy. From his constant activity and almost constant success in whatever he has undertaken, Sir William Collins could not fail to make his voice heard and his influence felt in the House of Commons. The part he has played in advancing scientific work, the encouragement he has given to the judicious support of the voluntary hospital system, and his steady and successful professional work at the Royal Eye and Temperance Hospitals and elsewhere, combine to make Sir William Collins eminently qualified for a seat in the House of Commons. Sir William has rendered such indisputable services to Local Government and Education in London that his election by any Metropolitan constituency should be a certainty, quite apart from his political opinions of which we can take no cognisance in The Hospital. Good men are scarce, talented men and men of robustness with wide experience in affairs, are all too few, and fewer still possess character and brains in equal proportions. All may surely be satisfied to cast their vote for the fortunate possessor of such talents, especially when he has given proof of his abilities, strength and character on many occasions, to the advantage of London and its inhabitants.

### What is Cancer?

In the Bradshaw Lecture delivered last week before the Royal College of Surgeons of England, Mr. H. T. Butlin chose as his text the positive assertion that "Carcinoma is a Parasitic Disease." There is nothing new in this as a general statement, for many pathologists have urged forward a similar hypothesis from time to time. However, largely because they have attempted to describe the actual parasite concerned, and their accounts have differed widely, they have failed to carry conviction. But Mr. Butlin makes no attempt to define or describe any particular parasite as the cause of cancer. Indeed he leaves it to conjecture whether the cancer parasite is an organism introduced from without or whether it is split off from the living cells of its host. Such an hypothesis may be regarded as the connecting link between the infective theory and Cohnheim's theory. Mr. Butlin's chief contention is that the carcinoma cell is an independent organism; that it lives a life which is wholly independent and proper to itself; and that it lives as a parasite in the body of the animal which is affected with carcinoma, deriving its nourishment from this host, and doing nothing to repay the host for the sustenance of which it robs him. Whatever truth there is in this statement the future alone can demonstrate. In the meantime Mr. Butlin will have performed a public service if he succeeds in giving vigour to a promising form of inquiry which has recently, and as we believe for insufficient reasons, become somewhat unpopular.

### Antivivisection Again,

Mr. Stephen Coleridge is active again. At a meeting of the Hospital Sunday Fund he moved amendments as the champion of the poor, amendments which his seconder, Archdeacon Wilberforce, attributed less to his love for the poor than to his hatred of the vivisector. This admission was not meant unkindly we believe. The amendments were lost, as they deserved to be, and if it be true that Mr. Stephen Coleridge and his party attempted to engineer a snap vote, the public will feel particularly glad that such unscrupulousness met with a fitting reward. The mental attitude of the antivivisector constitutes a psychological problem of some interest. The outstanding feature of that attitude appears to be an incapacity for that compromise which is an essential condition of life. Moderate people are prepared to estimate the permissibility of inflicting pain by the advantages to be derived from the infliction, and the measure of their consent is the measure of their belief that human life is more valuable than that of the lower animals. This may or may not be a defensible view, but it at least admits of a logical conclusion. The antivivisectionist, on the other hand, does not acknowledge this disparity of worth in the ranks of the creation; we should expect them all, therefore, to be vegetarian, at the least, and to abjure the use of any article of commerce which entails the slaughter, or even the temporary discomfort, of the brute creation. We strongly suspect that upon this account they should forswear the use of wool, for the newly-shorn sheep is probably much more to be pitied than the horse which supplies diphtheria antitoxin. Seriously, however, it is a distressing thing that kindness of heart should thus over-reach itself. Vivisection undoubtedly needs supervision; this supervision we have, and the penalties of abuse are stringently exacted. The uses of vivisection lie open to any eye that is not studiously blind.

